# Impact of Overweight and Obesity on Medical Care Costs, All-Cause Mortality, and the Risk of Cancer in Japan

**DOI:** 10.2188/jea.16.139

**Published:** 2006-07-13

**Authors:** Shinichi Kuriyama

**Affiliations:** 1 Division of Epidemiology, Department of Public Health and Forensic Medicine, Tohoku University Graduate School of Medicine.

**Keywords:** Neoplasms, Japan, Health Expenditures, Mortality, Obesity, Risk

## Abstract

We conducted three prospective cohort studies that examined the association between body mass index (BMI) and health outcomes in Japan. Our studies found statistically significant relationships between excess body weight and increased medical costs, all-cause mortality, and risk of cancer incidence. There was a U-shaped association between BMI and mean total costs. The estimated excess costs attributable to overweight and obesity was 3.2% of the total costs. This 3.2% is within the range reported in studies in Western countries (0.7%-6.8%). We observed statistically significant elevations in mortality risk in obese (BMI≥30.0kg/m^2^) women and lean (BMI<18.5 kg/m^2^) men and women. Our prospective cohort study found statistically significant relationships between excess weight and increased risk in women of all cancers. The population attributable fraction (PAF) of all incident cancers in this population that were attributable to overweight and obesity were 4.5% in women, which were within the range reported from Western populations, from 3.2% for US women to 8.8% for Spanish women. Our data suggests that excess body weight is a problem not only in Western countries but also in Japan.

Obesity is often considered to be a problem only in Western countries because of the low prevalence of obesity in Asian countries. Although obesity (body mass index [BMI] of 30.0 kg/m^2^ or higher) is less prevalent among Japanese (2-4%)^1^ than among Westerners (5-27%),^2^ epidemiologic studies have suggested that Japanese people may be more likely to develop adverse effects due to excess weight. For instance, the prevalence of type 2 diabetes, an obesity-related disease, is similar in Japanese (10-12%)^3^ and in Western (8-12%) populations,^4^^,^^5^ despite their differing proportions of obesity. It would therefore be necessary to examine the degree of adverse effects attributable to overweight and obesity in the Japanese population to fully understand the impact of excess weight upon societies worldwide.

This article is intended to introduce our three prospective cohort studies that examine the association between BMI and medical care costs, all-cause mortality, and the risk of cancer^6^^-^^8^ and to discuss the impact of obesity on health outcomes in Japan as compared with data from Western studies. In this paper, we classified ‘overweight’ as a BMI of 25.0 to 29.9 and ‘obesity’ as BMI of 30.0 or higher.

## COHORT STUDIES

### *1. Body mass index and medical care costs**^6^*

#### Methods

The data were derived from the Ohsaki National Health Insurance (NHI) beneficiaries cohort study. The subjects were all NHI beneficiaries, aged 40 to 79 years, living in the catchment area of Ohsaki Public Health Center, Miyagi Prefecture, northeast Japan. We prospectively collected data on medical care utilization and its costs for all individuals in the cohort between January 1995 and December 1998. The baseline survey was conducted between October and December 1994. Of 54,996 eligible individuals, 52,029 (95%) responded. We excluded 774 subjects because they had been withdrawn from the NHI before January 1, 1995, when we started prospective collection of NHI claim history files.

We excluded the following subjects from analysis: (1) those who did not provide information about body weight or body height; (2) those at both extremes of the BMI range: lower than the 0.05th percentile for BMI (below 14.41 for men; below 13.67 for women) or higher than the 99.95th percentile for BMI (above 58.46 for men; above 62.00 for women); (3) those who died before January 1, 1995 or within the first year during follow-up period; and (4) those who at baseline reported having had either cancer, myocardial infarction, stroke or kidney disease. Finally, we analyzed 41,967 subjects (20,067 men and 21,900 women).

The baseline survey included questions on body weight and body height, and BMI was calculated as the weight divided by the square of the height (kg/m^2^). When a beneficiary is withdrawn from NHI because of death or emigration, the data and reason are entered on the NHI withdrawal history files. Both NHI claims and withdrawal history files were linked with our baseline survey data files with a beneficiary ID number as a key code.

The impact of BMI on per month per capita total medical costs was examined by analysis of covariance. Per month values for each subject were calculated by dividing the accumulated values through observation by the number of months observed.

Excess medical costs of total health care expenditure attributable to overweight and obesity was calculated as follows:

A = (mean total costs for the overweight (BMI of 25.0 to 29.9) times the mean number of months observed among the overweight) minus (mean total costs at the nadir times the mean number of months observed at the nadir);
B = (mean total costs for the obese (BMI of 30.0 or higher) times the mean number of months observed among the obese) minus (mean total costs at the nadir times the mean number of months observed at the nadir);
C = A times the number of subjects who were overweight;
D = B times the number of subjects who were obese;

The proportion of excess medical costs of total health care expenditure attributable to overweight and obesity = (C + D) / total medical costs in the present study for four years. In this study, monetary values were converted into US dollars using the rate of US$ 1.00 = 120 Japanese yen.

#### Results

Table 1 shows adjusted per capita total medical costs by BMI levels. There was a U-shaped association between BMI and total medical costs. Medical costs were lowest at BMIs between 21.0 and 22.9. Relative to the nadir, mean total costs were 9.8% greater among the overweight (rate ratio, 1.10; 95% confidence interval [CI], 1.03 to 1.17), and 22.3% greater among the obese (rate ratio, 1.22; 95% CI, 1.08 to 1.37).

**Table 1.** Adjusted per capita per month total medical costs by body mass index levels.*

	Body Mass Index (kg/m^2^)						

	<18.5	18.5-20.9	21.0-22.9	23.0-24.9	25.0-29.9	≥30	P value
Total costs (US$)^†^	209.1	185.0	180.0	183.9	197.6	220.1	<0.001
95% confidence interval	193.0, 225.2	177.4, 192.6	174.0, 186.1	177.9, 189.9	191.6, 203.6	201.8, 238.3	

* : Published in ‘Kuriyama et al. Medical care expenditure associated with body mass index in Japan: the Ohsaki Study. Int J Obes Relat Metab Disord. 2002;26:1069-74’.

† : Adjusted for sex, age (continuous variable), smoking status (never, ever), alcohol drinking (currently drinking 46 gram or more ethanol a day, other) and physical functioning status (MOS scores 0-1, MOS scores 2-4, MOS scores 5-6).

The proportion of excess costs of total health care expenditure attributable to overweight and obesity in the present study = (C+D) / total medical costs in the present study for four years (US$ 360.8 million) = 0.032 (four fifths for overweight, one fifth for obesity).

### *2. Body mass index and all-cause mortality**^7^*

#### Methods

From June through August 1990, we delivered a self-administered questionnaire on various health habits to 51,921 subjects (25,279 men and 26,642 women) who were 40-64 years of age and lived in 14 municipalities of Miyagi Prefecture. Usable questionnaires were returned from 47,605 subjects (22,836 men and 24,769 women), yielding a response rate of 92%. We excluded subjects who indicated that they had prior histories of cancer, stroke, myocardial infarction, kidney disease, or liver disease. We also excluded subjects who had prevalent cancer at baseline. We further excluded subjects who had incomplete responses for body weight or body height information. Consequently, 39,610 subjects (18,740 men and 20,870 women) with 1,688 deaths (1,121 men and 567 women) were included in this analysis.

The baseline survey included questions on self-reports of body weight and body height. We followed up vital and residential status of subjects from June 1, 1990 through March 31, 2001. For this follow-up, we established the Follow-up Committee that was consisted of Miyagi Cancer Society. The Committee periodically reviewed the Residential Registration Record of each municipality. With this review, we identified the subjects who either died or emigrated during observation.

We counted person-years of follow-up for each subject from June 1, 1990 until the date of death, the date of emigration outside the study districts, or the end of the study period (March 31, 2001), whichever occurred first.

We used Cox proportional-hazards regression to estimate hazard ratios (HRs) as relative risks and 95% CI of all-cause mortality according to categories of BMI and to adjust for potentially confounding variables.

#### Results

Table 2 presents HRs for all-cause mortality according to categories of BMI. Age-adjusted analysis and multivariate analysis with or without the exclusion of subjects who died during the first three years of follow-up showed that there were statistically significant elevations in mortality risk in lean men and women and in obese women. Compared with a referent BMI group (23.0-24.9), multivariate HRs (95% CI) with the exclusion of deaths occurring in the first three years of follow-up were 2.06 (1.49-2.84) and 1.83 (1.17-2.88) for men and women, respectively, in the lowest BMI category (<18.5). The HR (95% CI) was 1.64 (1.09-2.49) for women in the highest BMI category (≥30.0). In contrast, HRs were not significantly increased or decreased among men across the BMI range of 18.5 or higher and among women across the BMI range of 18.5 through 29.9.

**Table 2.** Hazard ratios (HRs) and 95% confidence intervals (CIs) of all-cause mortality by body mass index categories in men and women.*

	Body Mass Index						

	<18.5	18.5-20.9	21.0-22.9	23.0-24.9	25.0-26.9	27.0-29.9	≥30
Men							
Person-years	3,603	30,136	51,932	55,414	31,107	17,137	3,770
No. of deaths	56	217	297	301	147	85	18
Age-adjusted HR (95%CI)	2.50 (1.88 - 3.33)	1.28 (1.07 - 1.52)	1.04 (0.89 - 1.22)	1.00	0.89 (0.73 - 1.08)	0.95 (0.75 - 1.21)	0.94 (0.59 - 1.52)
Multivariate HR1 (95% CI)^†^	2.05 (1.53 - 2.74)	1.10 (0.92 - 1.31)	0.97 (0.83 - 1.15)	1.00	0.92 (0.76 - 1.12)	1.01 (0.80 - 1.29)	1.00 (0.62 - 1.60)
Multivariate HR2 (95% CI)^‡^	2.06 (1.49 - 2.84)	1.12 (0.92 - 1.36)	0.98 (0.82 - 1.17)	1.00	0.96 (0.77 - 1.18)	1.05 (0.81 - 1.36)	0.85 (0.49 - 1.49)

Women							
Person-years	5,711	34,164	54,484	55,463	37,507	23,935	6,865
No. of deaths	29	75	126	144	95	64	34
Age-adjusted HR (95%CI)	1.96 (1.31 - 2.92)	0.94 (0.71 - 1.24)	0.95 (0.75 - 1.21)	1.00	0.90 (0.70 - 1.17)	0.95 (0.71 - 1.28)	1.72 (1.19 - 2.50)
Multivariate HR1 (95% CI)^†^	1.76 (1.16 - 2.67)	0.88 (0.66 - 1.17)	0.92 (0.72 - 1.17)	1.00	0.91 (0.70 - 1.17)	0.95 (0.70 - 1.27)	1.65 (1.13 - 2.40)
Multivariate HR2 (95% CI)^‡^	1.83 (1.17 - 2.88)	0.82 (0.60 - 1.14)	0.92 (0.70 - 1.19)	1.00	1.02 (0.78 - 1.34)	0.93 (0.67 - 1.28)	1.64 (1.09 - 2.49)

* : Published in ‘Kuriyama et al. Body mass index and mortality in Japan: the Miyagi Cohort Study. J Epidemiol 2004;14 (Suppl 1):S33-S8’.

† : Adjusted for age in years; weight loss of 5 kg or more since 20 years old (yes, no); marital status at baseline (whether or not living with spouse); cigarette smoking (never smokers, past smokers, current smokers smoking 1-19 cigarettes per day, or current smokers smoking at least 20 cigarettes per day); alcohol drinking (never drinkers, past drinkers, or current drinkers); walking time per day (less than 1 hour, or 1 hour or longer).

‡ : Multivariate HR2 has been estimated with the exclusion of 263 subjects (174 men and 89 women) who died within the first 3 years of follow-up.

### *3. Body mass index and the risk of cancer**^8^*

#### Methods

This prospective cohort study started in January 1984, when we delivered a self-administered questionnaire on various health habits to all residents 40 years of age or older (n=33,453) in three municipalities of Miyagi Prefecture. Usable questionnaires were returned from 31,345 subjects (17,353 women and 13,992 men; response rate 94%).

The self-administered questionnaire, which was used as a baseline survey, included questions on body weight and height. The end points in the analyses were incidence of all cancers (codes 140.0-195.8 and 199.0-208.9 of the International Classification of Diseases, 9th Revision [ICD-9]).^9^

We used population registries in the three municipalities to obtain data on the vital and residential status of the subjects from January 1, 1984 through December 31, 1992. We ascertained the incidence of cancer by means of computerized linkage with the records of the Miyagi Prefectural Cancer Registry. We documented a total of 2,646 cases of cancer among the subjects (1,156 women and 1,490 men). Of the 2,646 cases, we excluded the 541 (283 women and 258 men) that were diagnosed before the baseline survey.

We excluded subjects from the present analyses who did not answer the question about body weight or body height. We also excluded subjects with below-normal weight according to World Health Organization guidelines^10^ as indicated by a BMI of less than 18.5 to examine the dose response relationship between excess weight and cancer incidence. Consequently, our analysis included 27,539 subjects (15,054 women and 12,485 men) with a total of 1,672 cancer cases (668 women and 1,004 men).

We counted person-years of follow-up for each subject from January 1, 1984 until the date of diagnosis of cancer, the date of emigration outside the study districts, the date of death, or the end of the study period (December 31, 1992), whichever occurred first. We used the Cox proportional-hazards regression model to estimate HRs and 95% CIs of cancer incidence according to categories of BMI and to adjust for potentially confounding variables.

Because body weight is a modifiable risk factor, we calculated the population attributable fraction (PAF), an estimate of the proportion of all cancer incidences in Japan that might be avoided if the adult population maintained a BMI in the normal range. PAF was calculated as

*pd*×(HR－1) / HR,

where *pd* = proportion of cases exposed to the risk factor.^11^

#### Results

Table 3 lists the HRs of all cancers and the PAFs of all cancer incidences that were attributable to overweight and obesity. The multivariate HRs of all cancers associated with different BMIs, relative to a BMI of 18.5-24.9, were 1.04 (95% CI, 0.85-1.27) for BMI = 25.0-27.4, 1.29 (1.00-1.68) for BMI = 27.5-29.9, and 1.47 (1.06-2.05) for BMI ≥30.0 (P for trend, 0.007) in women. Although point estimates showed that BMI tended to have positive associations with the risk of all cancers, no statistically significant associations were observed in men overall. The PAFs of all cancer incidences that were attributable to overweight and obesity were 4.5% for women and -0.2% for men among all subjects. When HRs for never-smokers only were used, the PAF increased greatly, especially in men: 6.2% in women and 3.7% in men. The proportions of all cancer incidences attributable to excess weight were substantially modified by smoking status in this population.

**Table 3.** Hazard ratios (HRs) and their 95% confidence intervals (CIs), and population attributable fractions according to body mass index for cancer risk in Japanese women and men.*

Body Mass Index	Women			Men		
	
	Prevalence of cases^†^ %	HR^†^ (95%CI)	Population attributable fraction^‡^ %	Prevalence of cases^†^ %	HR^†^ (95%CI)	Population attributable fraction^‡^ %
All subjects						
25.0-29.9	26.2	1.12	2.8	18.2	0.97	-0.6
		(0.94-1.33)			(0.83-1.14)	
≥30.0	5.4	1.47	1.7	2.2	1.21	0.4
		(1.06-2.05)			(0.80-1.84)	
Total population attributable fraction			4.5			-0.2

Subjects who never smoked						
25.0-29.9	26.8	1.20	4.5	23.6	1.04	0.9
		(0.96-1.49)			(0.68-1.59)	
≥30.0	4.9	1.52	1.7	4.7	2.43	2.8
		(0.97-2.38)			(1.04-5.67)	
Total population attributable fraction			6.2			3.7

* : Published in ‘Kuriyama et al. Obesity and risk of cancer in Japan. Int J Cancer 2005;113:148-57’.

† : Data on prevalence of cases were caluculated including subjects with BMI of 18.4 or lower. Data on hazard ratios were from the results of the present study.

‡ : The population attributable fractions were calculated with the use of equation 5 in Rockhill et al.^11^

## DISCUSSION

We examined the relationship between BMI and medical care costs based on a prospective cohort of 41,967 Japanese adults aged 40 to 79 years over four years.^6^ The results demonstrated that there was a U-shaped association between BMI and mean total costs. We estimated the costs of overweight and obesity from the above results to be 3.2% of total costs. This 3.2% is within the range reported in studies in Western countries (0.7%-6.8%),^12^^-^^21^ although the definition of obesity varied among those studies (BMI of 25.0 or higher to BMI of 30.0 or higher).

Our prospective cohort study demonstrated that there were statistically significant elevations in mortality risk in obese (BMI≥30.0) women and lean (BMI<18.5) men and women.^7^ We did not observe significant differences in mortality for overweight subjects with BMI of 25.0 to 29.9 both in men and women. Although we could not explain this finding, the result is consistent with that from a large-scale study of 235,398 men in Korea, in which subjects with BMI of 24 to 29 had risks of death from 0.89 to 0.93 (reference; BMI of 22 to 23), which did not differ significantly from 1.00.^22^

Our prospective cohort study found statistically significant relationships between excess weight and increased risk in women of all cancers.^8^ The PAFs of all incident cancers in this population that were attributable to overweight and obesity were 4.5% (all subjects) or 6.2% (never-smokers) in women and -0.2% (all subjects) or 3.7% (never-smokers) in men. The results in women were within the range reported from Western populations, from 3.2% for US women^23^ to 8.8% for Spanish women.^24^ Despite the low prevalence of obesity in Japan, high relative risks of cancer yielded almost the same PAF in the Japanese population as in Western populations.

Less attention has been paid to excess weight as causes of adverse health outcomes in Japan than to other environmental factors, such as cigarette smoking, possibly because of the low prevalence of obesity. However, our prospective data demonstrate that the impact of overweight and obesity upon medical care costs, risk of cancer in women in Japan is as large as that of Western countries, suggesting that the Japanese are more likely to develop adverse effects due to slight excess weight. Our studies suggest that excess weight is now a problem not only in Western countries but also in Japan.
